# In Situ CO_2_ Efflux from Leaf Litter Layer Showed Large Temporal Variation Induced by Rapid Wetting and Drying Cycle

**DOI:** 10.1371/journal.pone.0108404

**Published:** 2014-10-01

**Authors:** Mioko Ataka, Yuji Kominami, Kenichi Yoshimura, Takafumi Miyama, Mayuko Jomura, Makoto Tani

**Affiliations:** 1 Laboratory of Forest Hydrology, Division of Environmental Science and Technology, Graduate School of Agriculture, Kyoto University, Kyoto, Japan; 2 Kansai Research Center, Forestry and Forest Products Research Institute (FFPRI), Kyoto, Japan; 3 College of Bioresource Sciences, Nihon University, Fujisawa, Kanagawa, Japan; DOE Pacific Northwest National Laboratory, United States of America

## Abstract

We performed continuous and manual in situ measurements of CO_2_ efflux from the leaf litter layer (*R*_LL_) and water content of the leaf litter layer (LWC) in conjunction with measurements of soil respiration (*R*_S_) and soil water content (SWC) in a temperate forest; our objectives were to evaluate the response of *R*_LL_ to rainfall events and to assess temporal variation in its contribution to *R*_S_. We measured *R*_LL_ in a treatment area from which all potential sources of CO_2_ except for the leaf litter layer were removed. Capacitance sensors were used to measure LWC. *R*_LL_ increased immediately after wetting of the leaf litter layer; peak *R*_LL_ values were observed during or one day after rainfall events and were up to 8.6-fold larger than *R*_LL_ prior to rainfall. *R*_LL_ declined to pre-wetting levels within 2–4 day after rainfall events and corresponded to decreasing LWC, indicating that annual *R*_LL_ is strongly influenced by precipitation. Temporal variation in the observed contribution of *R*_LL_ to *R*_S_ varied from nearly zero to 51%. Continuous in situ measurements of LWC and CO_2_ efflux from leaf litter only, combined with measurements of *R*_S_, can provide robust data to clarify the response of *R*_LL_ to rainfall events and its contribution to total *R*_S_.

## Introduction

Efflux of CO_2_ from the soil surface (soil respiration; *R*_S_), which is the sum of respiration by autotrophs and heterotrophs, is an important component of total CO_2_ efflux from forest ecosystems [1]–[3]. The *R*_S_: total ecosystem respirations varied from 58% to 76% in a mixed coniferous-deciduous forest [4], depending on interannual and seasonal changes in autotrophic and heterotrophic respiration; variability in *R*_S_ can affect the forest carbon balance on daily and seasonal time scales. To explain the cause of variability in *R*_S,_ many studies have attempted to separate differing sources of Rs and to examine factors controlling CO_2_ efflux rate from each source [5]–[7]. Especially in forest ecosystems, heterotrophic respiration consists of CO_2_ efflux from various sources (e.g., leaf and root litter, woody debris, soil organic matter) and their rates are controlled by their specific environmental condition such as water content (WC) and temperature [8], physical properties of the substrate (e.g., density and structure) [9], [10], and chemical properties (e.g., labile and recalcitrant carbon) [11], [12]. Moreover, CO_2_ efflux from the various heterotrophic sources responds differently to these controlling factors, which illustrates the complexity of *R*_S_. In recent decades, a variety of methods for separating components of heterotrophic respiration and for determining their contribution to total *R*_S_ have been developed [9], [13].

Among heterotrophic sources of CO_2_, the leaf litter layer (L-layer) is a significant reservoir of degradable carbon and a large potential source of CO_2_ efflux from forest soils [14]. In temperate forests, the contribution of CO_2_ efflux from the L-layer (leaf litter respiration; *R*_LL_) to *R*_S_ is reported to range from 23% to 48% [13], [15], [16]. The L-layer is in direct contact with rainfall, solar radiation, and wind, and environmental conditions (e.g., WC and temperature) can change more dynamically in the L-layer than in lower soil layers. Rapid and transient temporal variation in WC of the L-layer has been observed, especially in warm climates [16], [17]. Heterotrophic respiration responds rapidly to changes in moisture status [17], [18]; therefore, rapid and transient wetting and drying cycles would produce large temporal variations in *R*_LL_. This would significantly affect variation in *R*_S_ [17], [19], suggesting that *R*_LL_ is an important controller of temporal (daily and seasonal) patterns in the carbon balance in warm regions [19], [20].

Several methods for measuring *R*_LL_ and for calculating its contribution to *R*_S_ have been explored. Cisneros-Dozal et al. [21] used an isotope mass balance method and reported that the contribution of *R*_LL_ to *R*_S_ increased from 5% to 37% in response to water addition after transient drought. Deforest et al. [15] determined that the annual contribution of *R*_LL_ to *R*_S_ was 48% ±12% by measuring *R*_S_ with and without the L-layer, and the ratio was consistent over a range of environmental conditions. However, there is little information about temporal variation in *R*_LL_ in relation to rainfall events because of the difficulty of continuous and direct measurement of *R*_LL_ in situ.

To continuously measure CO_2_ efflux from the L-layer only, in parallel with measurement of *R*_S_, we developed an approach for measuring *R*_LL_ using an automated chamber method in a treatment area from which all CO_2_ sources except for the L-layer were removed. In parallel with *R*_LL_ and *R*_S_ measurements, we continuously measured water content of the L-layer (LWC) and soil water content (SWC). LWC was measured using a method developed by Ataka et al. [22], in which intact leaf litter was attached to surrounding capacitance sensors. Sensors were also placed on top of the L-layer and at the boundary between the L- and mineral layers. From these continuous in situ measurements, we investigated the response of *R*_LL_ to rainfall events by comparing *R*_LL_ with *R*_S_, and examined temporal variation in the contribution of *R*_LL_ to *R*_S_ in a warm temperate forest in Japan.

## Materials and Methods

### Ethics statement

The study site (Yamashiro Experimental Forest) is maintained by the Forestry and Forest Products Research Institute. All necessary permits were obtained for the field study, and the study did not involve endangered or protected species.

### Study site

Our observations of *R*_LL_ and *R*_S_ were conducted at the Yamashiro Experimental Forest in southern Kyoto Prefecture, Japan (34°47′N, 135°50′E). The study site is a 1.7-ha watershed characterized by an annual mean air temperature of 15.5°C (maximum, 34.8°C; minimum, −3.9°C) and annual precipitation of 1449 mm [2]. The rainy season generally occurs from early June to mid-July. Daily rates of evaporation from the forest floor are 0.4–0.8 mm day^–1^ for 1–2 days after precipitation, declining thereafter to 0.2–0.3 mm day^–1^ [23]. The soils are Regosols with sandy loam or loamy sand texture and contain fine gravel (53% by mass) composed of residual quartz crystals from granite parent material [24]. These are immature soils in which the thickness of the A horizon is 2–3 cm. Deciduous broad-leaved, evergreen broad-leaved, and coniferous tree species account for 66%, 28%, and 6% of the living tree biomass, respectively [25]. The forest is dominated by *Quercus serrata* Thunb., which accounts for approximately 33% of the biomass. The L-layer (approximately 3–4 cm thick) consists mainly of fresh *Q. serrata* litter. There is no substantial organic horizon below the L-layer.

### Automated chamber method for measuring leaf litter respiration and soil respiration

**Figure 1:**
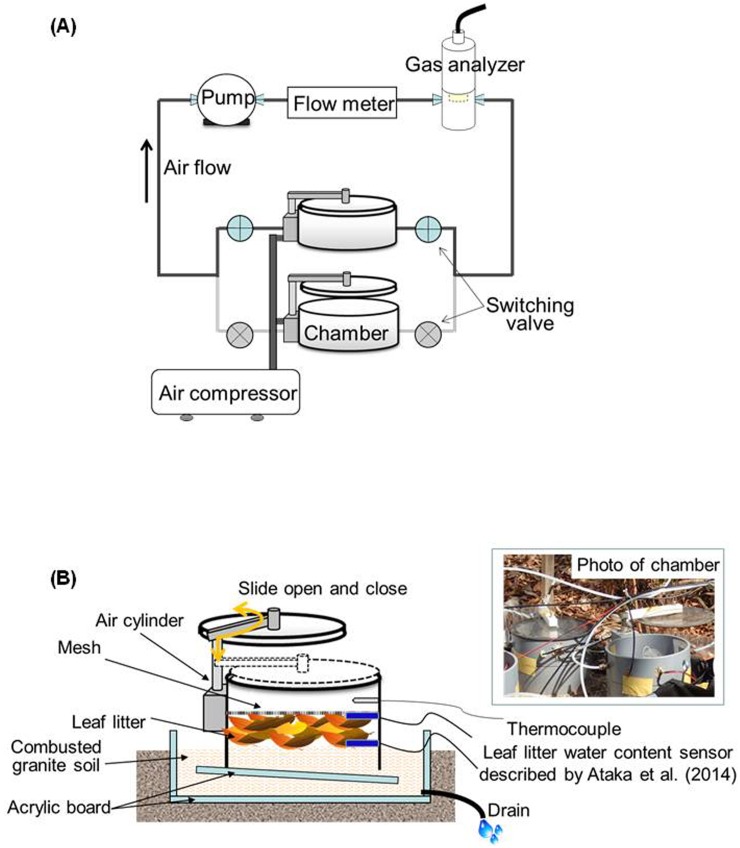
Schematic of the automated chamber system and the experimental design for measurement of CO_2_ efflux from the leaf litter layer. **A.** Schematic of the automated dynamic-closed chamber system for measuring leaf litter respiration and soil respiration. **B.** The experimental design for continuous measurement of CO_2_ efflux from the leaf litter layer only using automated chamber system.

We measured *R*_LL_ and *R*_S_ using an automated dynamic chamber system with an infrared gas analyzer (IRGA, GMP343; Vaisala Group, Vantaa, Finland) (Fig. 1A). The system consisted of two automated circular chambers for *R*_LL_ and *R*_S_ measurement, four solenoid valves, a pump, mass flow meter, and IRGA. The chambers (surface area 320 cm^2^) were made from PVC collars with clear acrylic lids that can be opened and closed automatically using an air cylinder. Air was supplied to the cylinder from a compressor. To ensure a seal between the chamber and the closed lid, a soft rubber gasket was attached to the top edge of the chamber. Opening and closing of the chamber lid and solenoid valves of each chamber were regulated synchronously by a control unit (ZEN, OMRON, Kyoto, Japan).

The duration of measurement of CO_2_ concentration inside each chamber was 6 min and was performed twice per hour. The CO_2_ concentration in each chamber was recorded at 1-s intervals using a data logger (GL220, Graphtec, Kanagawa, Japan). We calculated *R*_LL_ and *R*_S_ from the increase in CO_2_ concentration (ΔC_CO2_) using linear regression. Data from the first 2 min were discarded to avoid effects of closing the chamber. *R*_LL_ and *R*_S_ were calculated using the following equation:
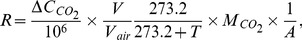
(1)where *R* is respiration (mg CO_2_ m^−2^ s^−1^), ΔC_CO2_ is the change in CO_2_ concentration per unit time (CO_2_ ppm s^−1^), *V* is the volume of the system (L), *V*_air_ is the standard gas volume (22.41 L mol^−1^), *T* is temperature inside the chamber (°C), *M*_CO2_ is the molecular weight of CO_2_ (44.01 g mol^−1^), and *A* is the soil surface area covered by the chamber (m^2^).

To continuously measure CO_2_ efflux from the L-layer only, we developed an approach for measuring *R*_LL_ by using an automated chamber method in a treatment area in which all potential CO_2_ sources (e.g., organic soil and fine roots) except for the L-layer were replaced with combusted granite soil (Fig. 1B). To prepare the treatment area (1 m^2^), we removed surface soil (approximately 5 cm). An acrylic board was placed on the bottom and sides of the treatment area to prevent penetration of roots; a drain tube was located at the bottom of the board to prevent the treatment area from flooding with rainwater. The treatment area was then filled with granite soil combusted in a muffle furnace (500°C for 1 day). For *R*_LL_ measurement, we placed a PVC collar (320-cm^2^ surface area) and acrylic board below the collar. The board was set at a slight incline to drain rainwater from the collar. We added 15 g of newly fallen leaf litter, which represents the average litterfall mass per unit ground surface area at this site, to the collar. We added the leaf litter to each chamber on January 2012. To acquire data on the temporal variation in *R*_LL_ of fresh leaf litter, we replaced the litter with newly fallen leaf litter in January 2013. The collar for measurement of *R*_S_ was placed near the treatment area for *R*_LL_ measurement and the L-layer inside the collar was removed and leaf litter was supplied similarly as for measurement of *R*_LL_. To prevent incorporation of newly fallen litter, we placed a mesh sheet (1×1 mm mesh) on the L-layer inside the chamber, and fallen litter was removed weekly. CO_2_ efflux from combusted granite soil was measured 6 months from the start of the *R*_LL_ measurements. The mean CO_2_ flux rate (± standard deviation) was 0.00063±0.00068 mg CO_2_ m^−2^ s^−1^ (*n* = 16) when SWC ranged from 0.05 to 0.3 m^3^ m^–3^ at temperatures of 24°C. Thus, we assumed that CO_2_ efflux from the combusted granite soil was negligible throughout the measurement period.

For continuous in situ measurement of LWC, we used capacitance sensors as described by Ataka et al. [22]. The measurements were performed on the top surface of the L-layer and at the boundary between the L-layer and mineral soil (Fig. 1B), to capture the large vertical distribution of WC within the L-layer. We estimated average LWC from the output voltage (V) of the two sensors using the conversion equation LWC = 12.73 V–3.42 presented by Ataka et al. [22]. LWC at the forest floor shows spatial variability associated with tree canopy conditions. Thus, to reflect the LWC of the L-layer by direct measurement, two capacitance sensors were placed on the L-layer inside the chamber. To check the validity of continuous LWC monitoring, we compared the sensor values with LWC measured manually as described in the following section. In parallel with LWC measurement, soil temperature (copper-constantan thermocouple) and soil volumetric water content (ECH_2_O EC-5 sensors; Decagon Devices, Pullman, WA, USA) were measured at 5-cm depth near each chamber. The output voltage of all environmental data was recorded every 1 min with a data logger (Datamark LS-3000 PtV; Hakusan, Japan) and average values were computed every 30 min. The environmental data, *R*_LL_, and *R*_S_ were measured continuously between September 2012 and January 2014. Malfunction of IRGA resulted in a lack of data for *R*_LL_ and *R*_S_ for 31% of the measurements.

### Manual chamber method for measuring leaf litter respiration and soil respiration

**Figure 2:**
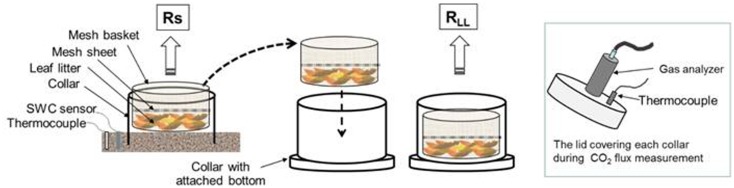
Schematic of the manual chamber system and the experimental design for measurement of CO_2_ efflux from the leaf litter layer (*R*_LL_) and soil (*R*_S_).

To determine the validity of *R*_LL_ and *R*_S_ measured using the automated chamber method, respiration was measured using the manual chamber method. We assumed that manual chamber method allow to measure under conditions that were closer to natural than the automated chamber method. We measured *R*_LL_ and *R*_S_ manually using a static chamber system at midday on 18 days between April 2013 and January 2014. Twelve PVC collars (320 cm^2^ surface area) were placed in a 2×4 m area in January 2013. The edges of the collars were inserted approximately 1.5 cm into the soil. To measure *R*_LL_, mesh baskets (1×1 mm mesh, the same diameter as the PVC collars; 20 cm) were set into each collar and 15 g (dry weight) of newly fallen leaf litter was placed on the L-layer inside each basket (Fig. 2). To prevent supply of newly fallen litter, we placed a mesh sheet (1×1 mm mesh) on the L-layer inside the chamber, and fallen litter was removed weekly.

For measurement of *R*_S_, the collars were completely covered with lids to which an IRGA and copper-constantan thermocouple were attached. Soil temperature and SWC (5 cm depth) were measured close to the collars when *R*_S_ was measured. After completing the measurements of *R*_S_, the mesh baskets were carefully removed from the collars and placed in PVC chambers (20 cm diameter, 7 cm high; Fig. 2). We measured *R*_LL_ using the same methods as used for *R*_S_ measurement. The temperature and CO_2_ concentrations in the chamber were recorded at 1-s intervals using a data logger (GL220). Linearity of the CO_2_ flux was checked on the data logger monitor at each measurement. The measurement period for each chamber was 10 min and CO_2_ data for the middle 5-min intervals were used to determine *R*_LL_ according to Eq. (1), excluding data from the first 3 min.

For measurement of LWC in the mesh baskets, four or five leaves were removed from each basket and immediately placed in sealed plastic bags. Fresh weight of the leaf litter was measured in the laboratory within 24 h of sampling. Leaf litter samples were oven dried at 65°C for 48 h, and water content (WC; g g^−1^) was calculated using Eq. 2 as follows:
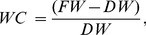
(2)where *FW* is the fresh mass of the sample (g), and *DW* is the dry mass of the sample (g). Samples were returned to each mesh basket within 1 week after sampling.

### Leaf litter respiration and soil respiration rates as a function of environmental factors

Respiration models are fundamentally described by nonlinear functions. We used the following function to investigate the response of respiration to temperature:
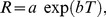
(3)where *T* is temperature (leaf litter temperature for *R*_LL_ measurement or soil temperature for *R*_S_ measurement) and *a* and *b* are constants. Leaf litter temperature was assumed to be same as air temprature. *b* is related to the Q_10_ parameter (Q_10_ = e^10b^). To determine the effects of temperature and water content on *R*_LL_ and *R*_S_, we used a function that was previously applied to estimate soil respiration by Subke and Schlesinger [26]:
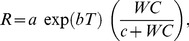
(4)where *a*, *b*, and *c* are constants. LWC or SWC was used as WC in this equation. These nonlinear regressions were performed using a modified Levenberg–Marquardt method with Igor Pro 6.0 software (WaveMetrics, Lake Oswego, OR, USA). The estimated respiration values presented in this manuscript were calculated using Eq. 4.

### Short-term changes in *R*_LL_ and LWC on wetting and drying cycle

To evaluate short-term changes in *R*_LL_ and LWC after rainfall events, we chose eight typical periods that included one wetting and drying cycle and had consecutive no rainfall days for at least 3 days. We used daily mean *R*_LL_ and LWC before the day on which precipitation occurred as the pre-wetting condition, and these values after precipitation as the post-wetting condition. Daily mean *R*_LL_ was calculated from *R*_LL_ values observed using the automated chamber method.

### Effect of wetting and drying cycle of the L-layer on *R*_LL_ and *R*s on the annual time scale

To investigate the effects of wetting and drying of the L-layer on *R*_LL_ on the annual time scale, we separated the estimated daily mean *R*_LL_ in 2013 into ‘Dry’ and ‘Wet’ periods based on daily mean LWC as a threshold value. The threshold LWC value that separated ‘Dry’ and ‘Wet’ periods for *R*_LL_ was estimated by the abovementioned short-term analyses. Daily mean *R*_LL_ was calculated from the estimated *R*_LL_ values because there were gaps in the continuous *R*_LL_ data observed using the automated chambers. We estimated the contribution of *R*_LL_ accumulated during the wet and dry period to total *R*_S_.

## Results

### Seasonal variation in *R*_LL_ and *R*_S_

**Figure 3:**
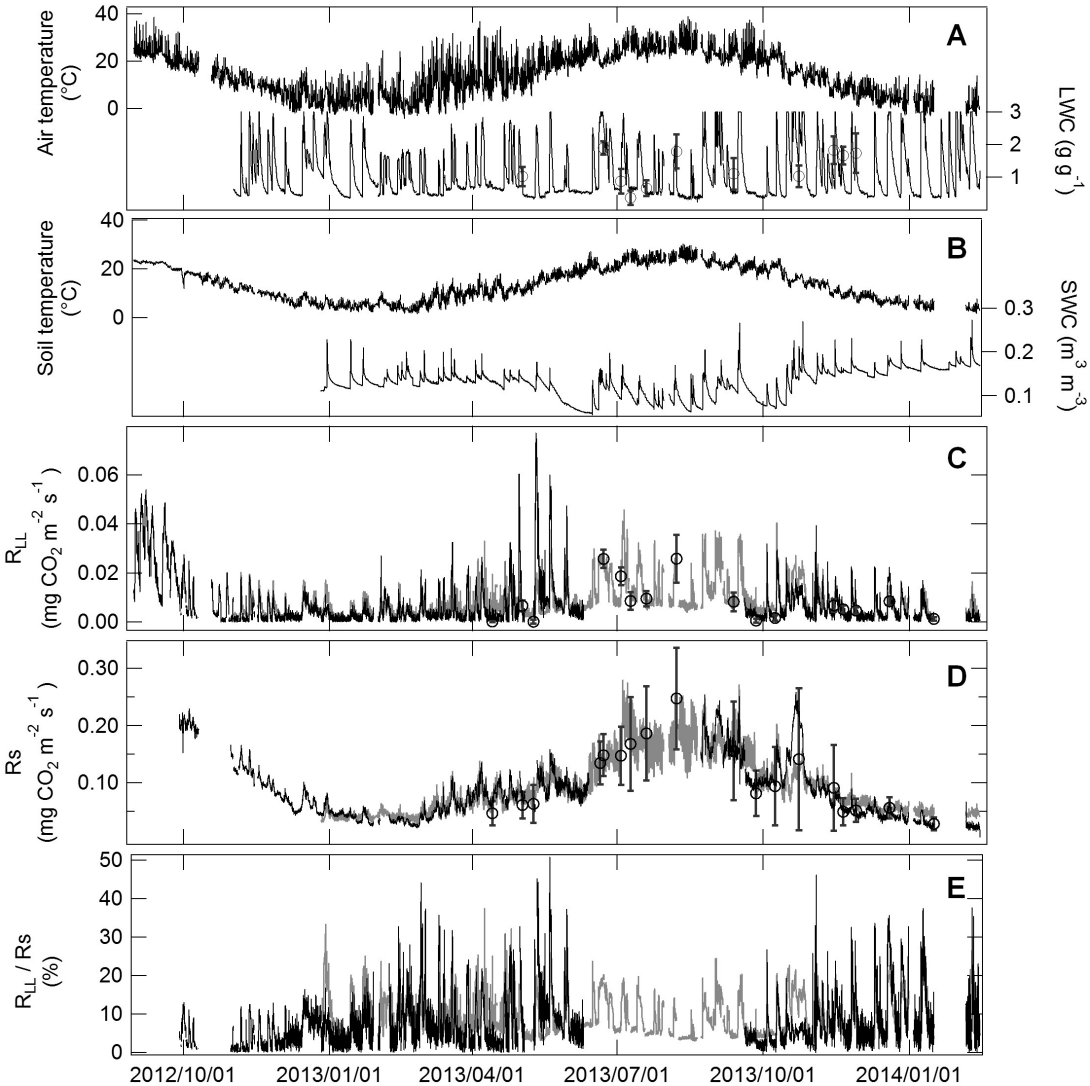
Seasonal variation in environmental factors, CO_2_ efflux from the leaf litter layer (*R*_LL_), and soil respiration (*R*_S_). Data were measured every 30 min between September 2012 and January 2014. **A.** Bold and fine lines show air temperature and water content of the leaf litter layer (LWC), respectively. **B.** Bold and fine lines show soil temperature and soil water content (SWC), respectively. **C.** Black and grey lines show observed and estimated *R*_LL_, respectively. **D.** Black and grey lines show observed and estimated *R*_S_, respectively. **E.** Black and grey lines show the ratio of observed and estimated *R*_LL_ to *R*_S_, respectively. Circles and bars show mean values and standard deviation of manual measurements. Estimated *R*_LL_ and *R*_S_ were calculated from regression equations using temperature (T) and water content (WC): *R*_LL_ = 0.29e^0.059T^[WC/(95.04+WC)] and *R*_S_ = 0.031e^0.10T^[WC/(0.032+WC)].

The magnitude of the peak in the observed *R*_LL_ pulse was higher in summer than in winter (Fig. 3C). *R*_LL_ values were low when LWC was low (Fig. 3A, C). *R*_S_ changed substantially according to temperature (Fig. 3B, D), with higher values in summer than in winter. The relationships between respiration and temperature were described by the following functions:
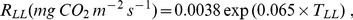
(5)

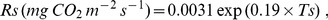
(6)where *T*_LL_ is leaf litter temperature and *T*s is soil temperature (°C). To evaluate effect of WC on the temperature sensitivity of respiration, the measured respiration data was separated into three groups based on WC (Table 1). More than 14% of total respiration data was included in each WC group. *R*_LL_ showed low values when WC values were low in spite of high temperature. Consequently, calculated Q_10_ values for not only *R*_LL_ but also *R*_S_ decreased with decreasing WC. The relationships between respiration and temperature and WC were described by the following functions:

**Table 1:** Q_10_ of leaf litter respiration (*R*_LL_) and soil respiration (*R*_s_) for different water contents of the leaf litter layer (LWC) and soil (SWC).

	*R*_LL_			*R*_s_		
	LWC≤1	1<LWC≤2	2<LWC	SWC≤0.1	0.1<SWC≤0.15	0.15<SWC
**Q_10_**	1.54	1.88	2.07	1.97	2.12	2.73
**a**	0.0019	0.0044	0.0064	0.027	0.032	0.025
**b**	0.043	0.063	0.073	0.068	0.075	0.10



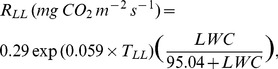
(7)

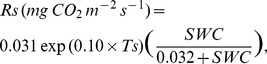
(8) where LWC (g g^−1^) and SWC (m^3^ m^−3^) are water content of leaf litter and soil, respectively. The RMSE between observed and estimated daily mean respiration based on temperature (*R*_LL_, 0.0080 mg CO_2_ m^−2^ s^−1^; *R*_S_, 0.060 mg CO_2_ m^−2^ s^−1^) was larger than that based on temperature and WC (*R*_LL_, 0.0046 mg CO_2_ m^−2^ s^−1^; *R*_S_, 0.012****mg CO_2****_m^−2****^s^−1^) (Fig. 4). Estimated respiration was calculated using the equation based on temperature and WC because of the lower RMSE. Throughout the measurement period, the contribution of observed *R*_LL_ to variation in *R*_S_ changed from nearly zero to 51% following a rainfall event (Fig. 3E).

**Figure 4:**
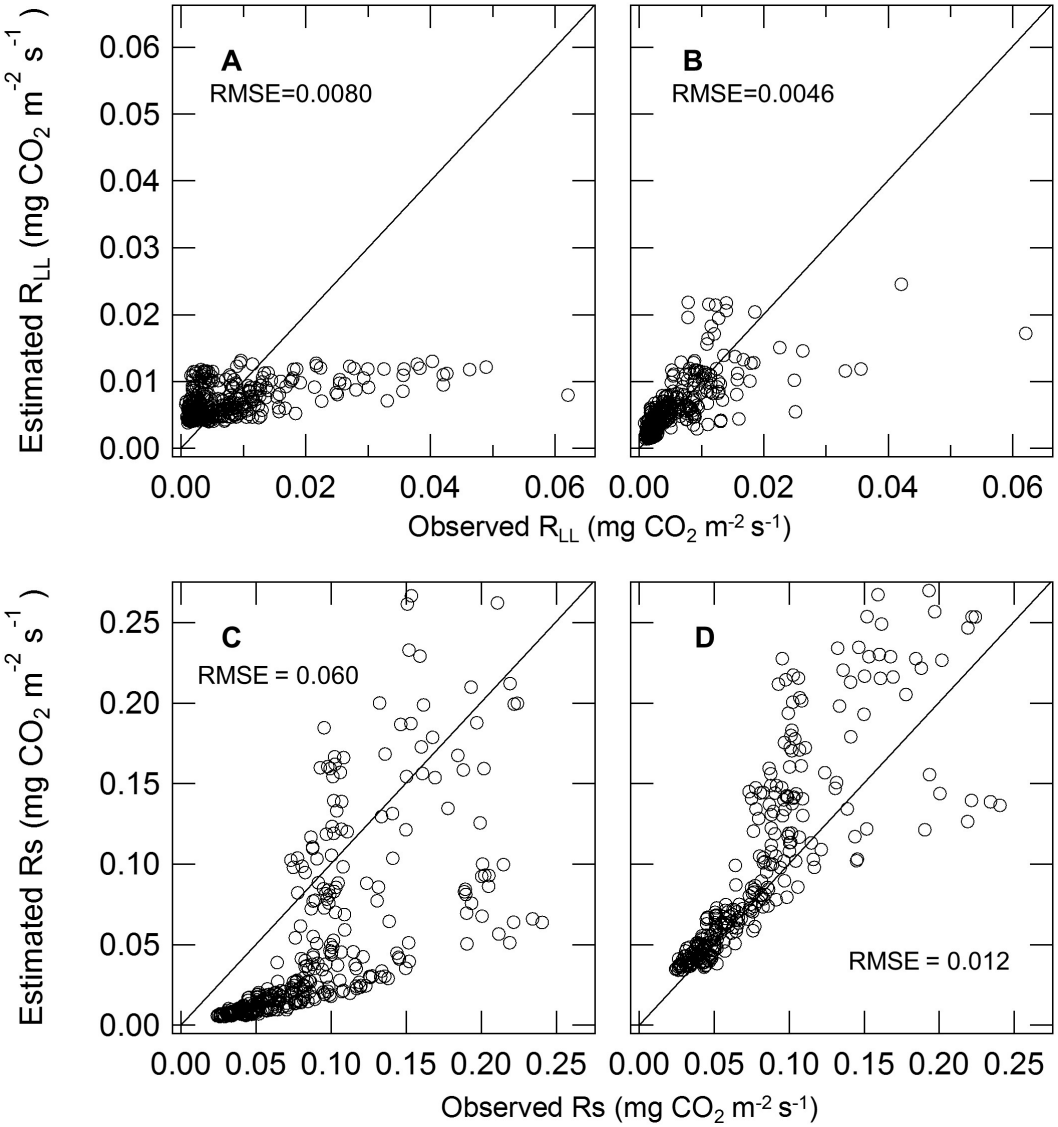
Relationship between observed and estimated CO_2_ efflux rate from leaf litter respiration (*R*_LL_) and soil respiration (*R*_S_). *R*_LL_ (A, B) and *R*s (C, D) show daily mean values. Estimated respiration rates were calculated using a function of temperature (A, C) from Eq. (5,6) and a function of temperature and water content (B, D) from Eq. (7,8) in the Results. Lines represent the 1∶1 ratio. RMSE: root mean square error.

**Figure 5:**
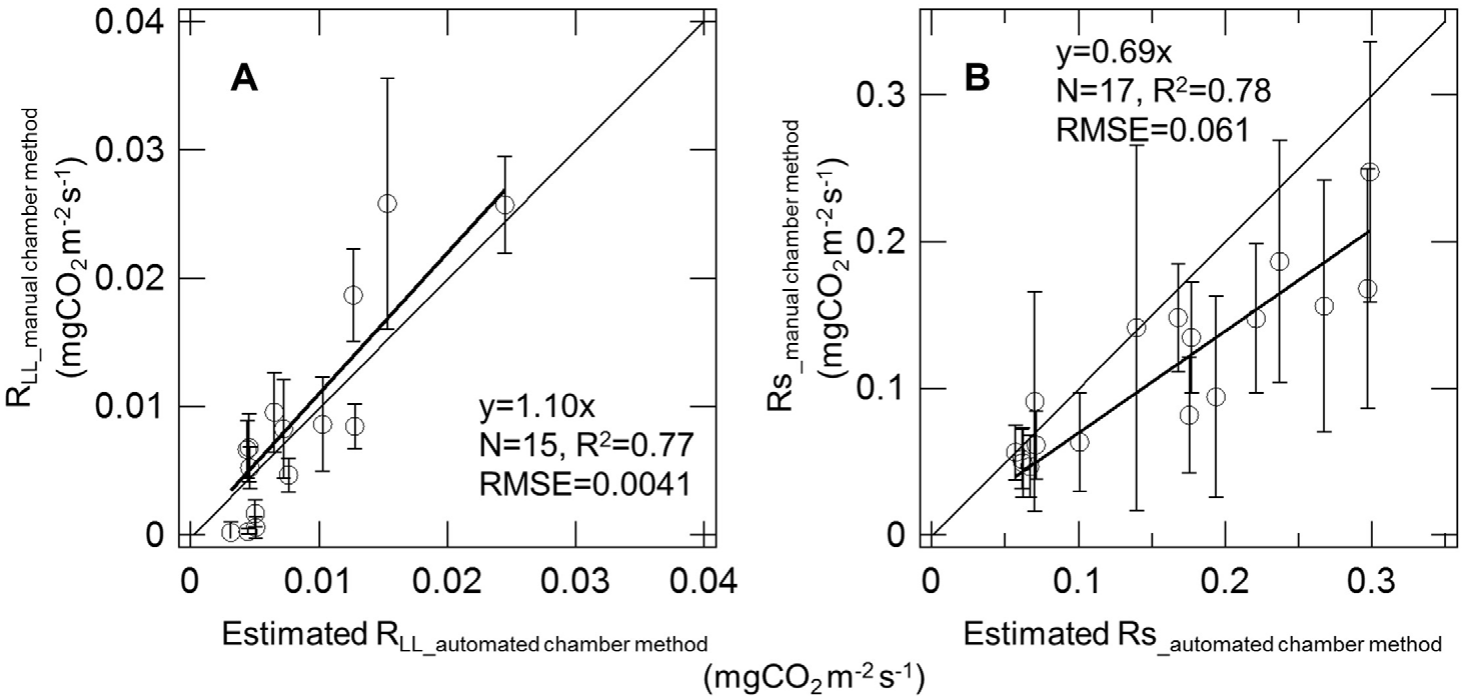
Relationship between respirations measured using a manual chamber method and estimated from automated chamber data. Respiration rate measured with the manual chamber method (*R*__manual chamber method_) show mean value obtained from measurement of 12 collars. Bars show standard deviation. Respiration estimated from automated chamber data (estimated *R*__automated chamber method_) shows daily mean respiration. The estimated *R* was calculated using a function based on temperature and water content (Eq. 8, 9).

To consider the validity of *R*_LL_ and *R*_S_ estimated from continuous measurement, we compared these values with respiration rates measured using the manual chamber method (Fig. 5). Estimated respiration was very similar to that observed using manual measurements. The RMSE between estimated and observed respiration were 0.0041 and 0.061****mg CO_2****_m^−2****^s^−1^ for *R*_LL_ and *R*_S_, respectively.

### Temporal changes in *R*_LL_ and *R*_S_ on the short-term scale

**Figure 6:**
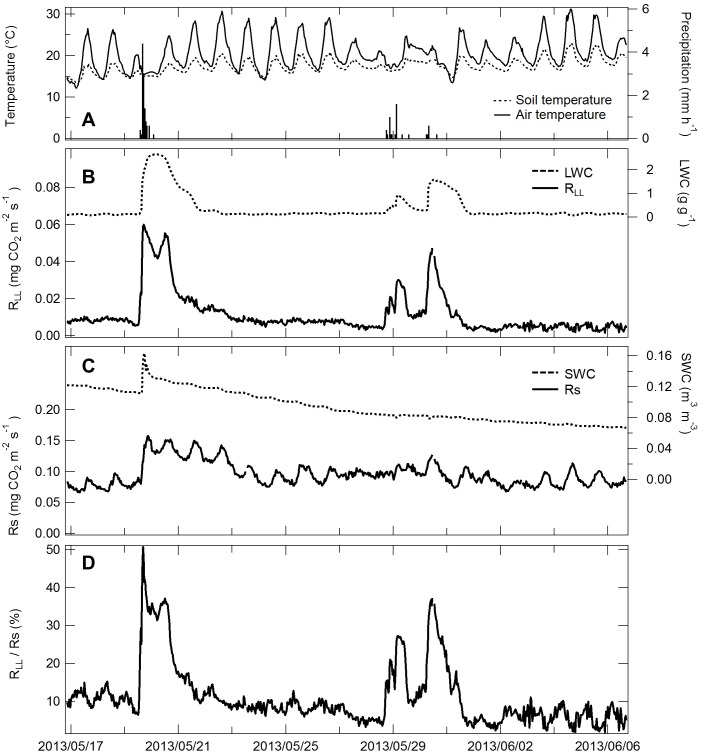
Temporal variation in environmental factors, CO_2_ efflux from the leaf litter layer (*R*_LL_), soil respiration (*R*_S_), and the ratio of *R*_LL_ to *R*_S_. Data was measured at one collar every 30 min between May 17 and June 6, 2013. **A.** Soil and air temperature. Spikes on the x-axis indicate precipitation events (mm h^−1^). **B.** *R*_LL_ and water content of the leaf litter layer (LWC). **C.** *R*_S_ and soil water content (SWC). **D.** The ratio of *R*_LL_ to *R*_S_ (%).

To show clear temporal variation in *R*_LL_ and *R*_S_, the period between May 17 and June 6, 2013 (Fig. 6) was chosen because this period included two characteristic rainfall events. The rainfall intensity was 11.6****mm over 13****h during the first event and 5.4****mm over 46 h during the second event. LWC and SWC increased from 0.11 to 2.64 g g^−1^ and from 0.11 to 0.16 m^3^ m^−3^, respectively, following the first rainfall event (Fig. 6B, C). LWC increased from 0.16 to 1.58 g g^−1^ but SWC did not increase after the second rainfall event.

Temporal variation in *R*_LL_ measured using the automated chamber system changed according to wetting and drying of the L-layer (Fig. 6B), reaching a maximum of 0.060 and 0.047 mg CO_2_ m^−2^ s^−1^ during first and second rainfall events, respectively. *R*_S_ increased following the increase in SWC and subsequently decreased gradually with diurnal variation according to temperature (Fig. 6C). Between May 17 and June 6, 2013, the contribution of *R*_LL_ to *R*_S_ increased from 6.5% to 51%, with a peak value of 51% during the first rainfall event and 37% during the second rainfall event (Fig. 6D).

**Figure 7:**
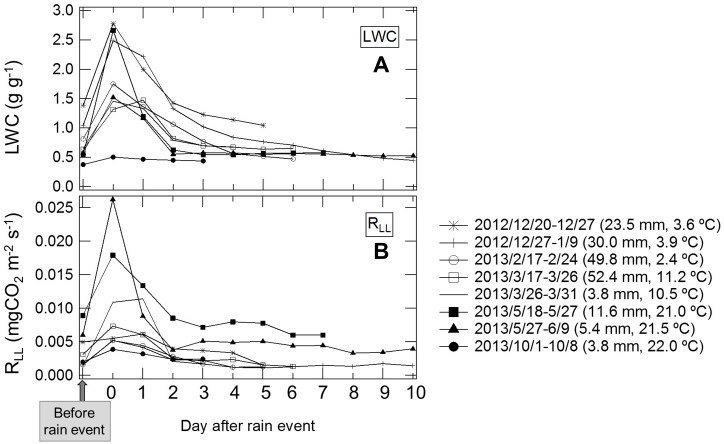
Temporal variation in water content of the leaf litter layer (LWC) and CO_2_ efflux from the leaf litter layer (*R*_LL_) after rainfall events. LWC (A) and *R*_LL_ (B) show the daily mean values. The rainfall intensity of each precipitation event was 23.5 mm in 2 days (2012/12/20–12/27, mean air temperature; 3.6°C); 30.0 mm in 3 days (2012/12/27–1/9, 3.9°C); 49.8 mm in 2 days (2013/2/17–2/24, 2.4°C); 52.4 mm in 3 days (2013/3/17–3/26, 11.2°C); 3.8 mm in 2 days (2013/3/26–3/31, 10.5°C); 11.6 mm in 2 days (2013/5/18–5/27, 21.0°C); 5.4 mm in 3 days (2013/5/27–6/9, 21.5°C); and 3.8 mm in 4 days (2013/10/1–10/8, 22.0°C).

Both *R*_LL_ and LWC reached a peak during or one day after rainfall events (Fig. 7). The peak of *R*_LL_ and LWC varied from 0.0020 to 0.026 mg CO_2_ m^−2^ s^−1^ and from 0.50 to 2.66 g g^−1^, respectively. Peak value of each rainfall event highly depended on air temperature. High peaks of *R*_LL_ were observed in the warm season (0.017 mg CO_2_ m^−2^ s^−1^; 2013/5/18–5/27, 0.026 mg CO_2_ m^−2^ s^−1^; 2013/5/27–6/9 in Fig. 7). Also, the peak value was related to LWC: low peak of *R*_LL_ was observed when LWC was low (0.004 mg CO_2_ m^−2^ s^−1^; 2013/10/1–10/8 in Fig. 7). The relationship between LWC and amout of precipitation was not clear. In the cold season, peak values of *R*_LL_ were relatively low (e.g., 0.005 mg CO_2_ m^−2^ s^−1^; 2013/2/17–2/24, 0.006 mg CO_2_ m^−2^ s^−1^; 2012/12/20–12/27 in Fig. 7) even when the L-layer was wet enough (LWC more than 1.5 g g^−1^). The peak values of *R*_LL_ were 1.2- to 8.6-fold higher than the *R*_LL_ values before rainfall events, and *R*_LL_ fell to pre-wetting levels within 2–4 days after rainfall events and peak LWC values were 1.3- to five-fold higher than LWC before rainfall, and LWC also dropped to pre-wetting levels within 2–4 days after rainfall events.We defined *R*_LL_ from the period just after rainfall events through 2–4 days later as the “*R*_LL_ pulse”.

### Effects of wetting and drying of the L-layer on *R*_LL_ and *R*_S_ on the annual time scale

**Figure 8:**
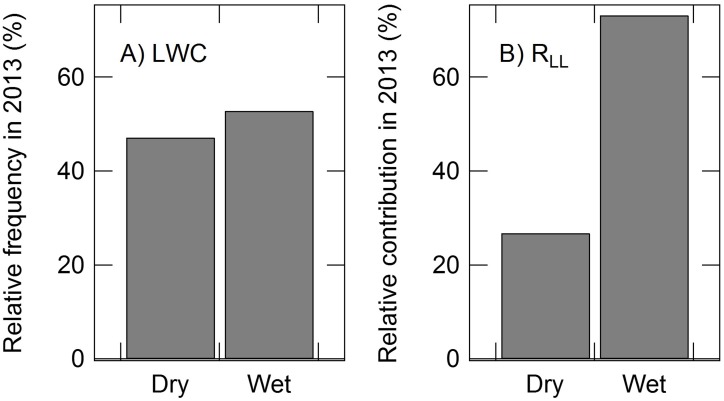
Histograms of the relative frequency of “Dry” and “Wet” periods in relation to water content of the leaf litter layer (LWC), and the relative contribution of estimated leaf litter respiration (*R*_LL_) in 2013. The daily mean LWC (A) and *R*_LL_ (B) were used to present histograms. Estimated respiration rates were calculated using a function based on temperature (T) and water content (WC). *R*_LL_ = 0.29e^0.059T^[WC/(95.04+WC)]. The daily mean LWC and *R*_LL_ were defined as Dry or Wet based on LWC. Days in which daily mean LWC <0.75 g g^−1^ were defined as Dry periods, while days in which daily mean LWC ≥0.75 g g^−1^ were defined as Wet periods.

Estimated daily mean *R*_LL_ in 2013 was separated into ‘Dry’ and ‘Wet’ periods based on daily mean LWC. Days for which mean LWC was <0.75 g g^−1^ were categorized as Dry, while days for which mean LWC ≥0.75 g g^−1^ were categorized as Wet. The threshold value (0.75 g g^−1^) was obtained from mean LWC 3 days after a rainfall event (Fig. 7A). The relative frequency of Dry and Wet periods in 2013 were 47.2% and 52.8%, respectively, while the relative contributions of daily mean *R*_LL_ during the Dry and Wet periods in 2013 were 26.9% and 73.2%, respectively (Fig. 8). Annual *R*_LL_ and *R*_S_ in 2013 were estimated to be 0.69 and 7.94 t C ha^−1^ y^−1^, respectively. The RMSE between continuous respiration measured and estimated based on temperature and WC was 0.011 and 0.029 t C ha^−1^ y^−1^, respectively.

The contribution of annual *R*_LL_ to *R*_S_ was 8.6%. The relative frequency of LWC was similar during Dry and Wet periods, while the contribution of *R*_LL_ during the Wet period was approximately three-fold higher than that during the Dry period (Fig. 8).

## Discussion

As seen in Fig. 6, *R*_LL_ immediately increased with wetting of the L-layer and decreased to pre-wetting levels within 2–4 days after rainfall events, which was consistent with observations made in previous studies [17], [19]. *R*_LL_ showed no diurnal variation despite a diurnal temperate range >10°C. Consequently, the Q_10_ of *R*_LL_ increased with increasing LWC (Table. 1). The variation in Q_10_ would be directly related to water stress experienced by microorganism. This indicated that LWC can reach to adequate low value, suspected as water stress for microorganism, within several days after rainfall. On the one hand, *R*_S_ increased during rainfall and subsequently decreased, showing diurnal variation. The Q_10_ of *R*_S_ also increased with increasing SWC. Dannoura et al. [27] reported that root respiration showed little change with variation in SWC compared with changes in *R*_S_. Therefore, the increased Q_10_ of *R*_S_ with increasing SWC might be highly affected by not only *R*_LL_ but also by respiration from other heterotrophic sources.

Although the relative frequency of LWC was similar during Dry and Wet periods, the contribution of annual *R*_LL_ during the Wet period was approximately three-fold higher than that during the Dry period (Fig. 8), indicating strong effect of rainfall on *R*_LL_. Although the *R*_LL_ pulse can last for only 3–4 days after a rainfall event, this pulse would determine a large part of annual *R*_LL_. This suggests that the magnitude of total *R*_LL_ may be influenced by the frequency of rainfall events, especially in summertime, rather than the intensity of rainfall. Still, the cumulative *R*_LL_ in the Dry period contributed 26.9% of annual *R*_LL_ in 2013, even though instantaneous *R*_LL_ was very low. There may be large vertical variability in WC and *R*_LL_ within the L-layer, indicating that higher WC and *R*_LL_ occur in lower parts of the L-layer during the drying process because the upper L-layer dries more rapidly [28]. In that case, although the mean WC of the L-layer was very low, local wetting in lower sections would produce small CO_2_ fluxes. Despite low instantaneous *R*_LL_, the accumulation of *R*_LL_ over a long time period (approximately 6 mo) resulted in a substantial contribution (27%) of Dry-period respiration to annual *R*_S_.

Raindrops first reach the L-layer and then percolate to the soil layers below. Small amounts of precipitation caused no change in SWC or *R*_S_, but *R*_LL_ increased rapidly with increasing LWC (Fig. 6). In semi-arid and arid ecosystems, wetting of the L-layer and surface soil by small fog-drop pulses during the dry season can contribute up to 35% of *R*_S_ [29]. Although such small water inputs (e.g., brief rain showers and fog), which mainly affect the surface of the forest floor, can be significant drivers of temporal variation in *R*_S_, the soil water content sensors (generally inserted at depths >5 cm) could not capture these inputs. Continuous measurement of LWC allowed for realistic modeling of the effects of rapid changes in LWC on *R*_LL_.

Although the annual contribution of *R*_LL_ to *R*_S_ was relatively small (8.6%), this contribution showed large temporal variation according to rainfall, ranging from nearly zero to 51%. Several other studies have described similar results [17], [21]. For example, Borken et al. [17] reported that peaks in *R*_LL_ during addition of water ranged from 0.031 to 0.071 mg CO_2_ m^−2^ s^−1^ in vitro, which represented 11–26% of maximum in situ *R*_S_ in the Harvard forest, although *R*_LL_ before addition of water was nearly zero. These findings indicate that *R*_LL_ is a significant component of rapid and transient temporal variation in *R*_S_ in relation to rainfall events. Although numerous studies have examined CO_2_ efflux from mineral soils in relation to the intensity, duration, and frequency of rainfall [30], [31], few studies have focused on *R*_LL_ because of the difficulty in measuring this dynamic. Here, *R*_LL_ pulses were observed only during and several days after rainfall events. Thus, periodic sampling (e.g., twice per week) might be insufficient to capture the contribution of the *R*_LL_ pulse to *R*_S_. Moreover, manual flux measurements are usually not performed during precipitation events because of difficulties that can occur with electronic instruments and sampling methods. In our view, conducting in situ measurements of CO_2_ efflux from the L-layer only over short time intervals (e.g., up to 1 h) produces robust data for understanding the response of *R*_LL_ to rainfall events and its contribution to *R*_S_.

The contribution of *R*_LL_ to annual *R*_S_ was 8.6% in our site. In an oak forest, the contribution of *R*_LL_ to *R*_S_ was 23%, according to model simulation based on temperature and LWC by Hanson et al. [13]. Ngao et al. [32] reported a lower contribution (8%) in a beech forest, estimated using an isotope mass balance approach, which was close to the value observed at our site (8.6%). However, simple quantitative comparisons between studies are difficult because of the use of different methods. In addition, some technical problems remain at our site. First, we performed *R*_LL_ measurements in the treatment area in which the mineral soil below the L-layer was replaced with combusted granite soil. This treatment may have affected the microbial community and environmental conditions in the L-layer. Secondly, each continuous measurement of *R*_LL_ and *R*_S_ was performed with single chambers, so spatial heterogeneity in *R*_LL_ and *R*_S_ were not considered. Automated chamber methods allowed high-interval measurements of temporal variation in respiration but had poorer spatial distribution compared with the manual chamber method. The balance of trade-offs between automated and manual chamber method is subject to the relative importance of characterizing temporal and spatial variability of individual CO_2_ sources. The number of chambers used can enhance the accuracy of measured mean values. Loescher et al. [33] reported that the number of chambers needs to be >100 to adequately represent spatial variability. However, this is not a feasible experimental design because of practical limitations to sampling efforts. To improve estimation of *R*_LL_ and *R*_S_ at the forest stand level, and to better understand the soil carbon budget, a comprehensive comparison of the diverse C pools and fluxes in forest soils is required.

## Conclusions

In our study, the rapid and transient variation in *R*_LL_ induced by rainfall; the peak *R*_LL_ was observed during or one day after rainfall, and *R*_LL_ subsequently decreased to pre-wetting levels within 2–4 days after rainfall events, following the decrease in LWC. On the one hand, CO_2_ efflux from coarse woody debris found in our site decreased during rainfall events, and subsequently, a gradual increase in CO_2_ efflux continued for at least 14 days until next rainfall [34]. Therefore, coarse woody debris was a CO_2_ efflux source over longer time scales, while *R*_LL_ approached nearly zero within a few days after rainfall events, even at high temperatures. Such specific temporal CO_2_ efflux patterns for each heterotrophic source when subjected to wetting and drying cycles would be a result of substrate properties (e.g., specific surface area). In our view, continuous and direct measurements of CO_2_ efflux and environmental conditions characterized by substrate properties of individual CO_2_ sources could improve understanding of the processes that regulate variation in heterotrophic respiration and *R*_S_ and enable progress beyond empirical models that are primarily based on simple temperature and SWC relationships.

Moreover, the magnitude of heterotrophic respiration under wetting and drying cycles is strongly related to microbial physiology and community composition. For example, Schnurer et al. [35] showed that longer-duration wetting could promote microbial biomass, causing an increase in basal respiration. Fierer et al. [36] showed the influence of drying and rewetting frequency on microbial (fungi and bacteria) community composition. To improve understanding of heterotrophic respiration associated with response and adaptation of microorganisms under climatic changes, collected continuous in situ data for CO_2_ efflux and environmental conditions (e.g., temperature and WC) of individual CO_2_ sources should be combined with analyses of microbial physiology and community composition.
